# Projecting changes in extreme rainfall from three tropical cyclones using the design-rainfall approach

**Published:** 2021-03-25

**Authors:** Anna M. Jalowska, Tanya L. Spero, Jared H. Bowden

**Affiliations:** 1 Office of Research and Development, U.S. Environmental Protection Agency, Research Triangle Park, NC, USA; 2 Department of Applied Ecology, North Carolina State University, Raleigh, NC, USA

## Abstract

In the past quarter-century, Eastern North Carolina (ENC) experienced several devastating tropical cyclones that led to widespread flooding and damage. Historical climate records reflect an increasing trend in the frequency and intensity of extreme rainfall events across the eastern U.S., which is projected to continue to increase throughout the twenty-first century. Potential changes to extreme rainfall across ENC are explored and quantified for 2025–2100 for three tropical cyclones using an approach based on relative changes in future extreme rainfall frequencies (return periods) from dynamically downscaled projections. Maximum rainfall intensities at ‘2100’ could increase locally by 168%, with widespread regional increases in total rainfall up to 44%. Although these magnitudes exceed the consensus in the literature, the values here are comparable to the most extreme rainfall events observed in the U.S. during the early twenty-first century, which suggests that the intensity of projected future events is already a present-day reality.

## INTRODUCTION

Extreme precipitation events driven by tropical cyclones (TCs) disrupt the quality of life in coastal plains and low-lying communities, where growing populations support important agricultural, environmental, cultural, and economic resources. Consequently, there is a broad interest in quantifying the potential changes to extreme rainfall from TCs. Over the past century, the southeastern U.S. has increasingly recorded more extreme precipitation events^1^. Since 1910, much of the U.S. has received a greater proportion of its total precipitation from intense one-day rainfall events^2^. During 1958–2016, there was a 27% increase in the 99th percentile precipitation^3^ in the southeastern U.S. During 1949–2018, TCs comprised 25 of the top 100, 4-day rainfall events nationwide^4^; with one-quarter of the top 100 events occurring in the final decade of the 70-yr record, at a far greater frequency than in any other decade. Throughout the mid-latitudes, extreme rainfall events will likely become more intense in a warmer world from increased tropospheric water vapor^5^. The 20-yr return value of the annual maximum daily rainfall—a measure often used in urban planning—could increase by an average of 5.3% °C^−1^ warming with wide regional variability^6^. TC precipitation will likely increase globally, and a 2 °C warming could increase the median TC precipitation rate by 14% at constant relative humidity^7^.

**Fig. 1:**
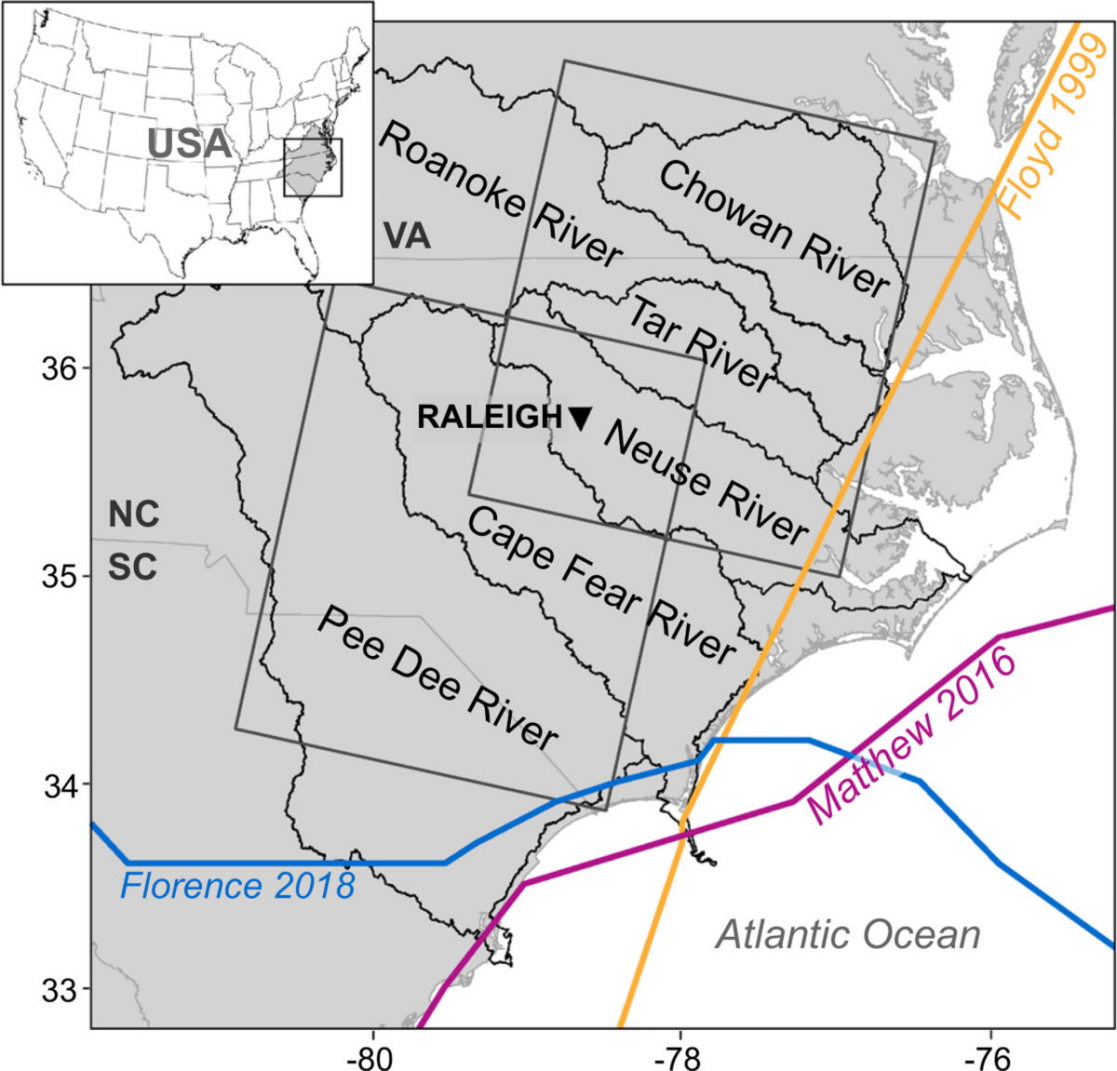
ENC study area. Inset shows the location of Eastern North Carolina (ENC) within the United States. The primary image shows the six ENC watersheds considered in this study (labeled within amorphous black outlines). State boundaries are denoted with medium-gray outlines, where VA is Virginia, NC is North Carolina, and SC is South Carolina. The tracks of Floyd (yellow), Matthew (violet), and Florence (blue) are superimposed. Boxes denoted with dark gray lines correspond to the 2° × 2° (50,000 km^2^) area used in Kunkel & Champion^4^. The box to the northeast was used to derive the average intensity (AI) for Floyd and Matthew, and the box to the southwest was used for Florence.

Eastern North Carolina (ENC, Fig. 1) is among the most vulnerable regions for TC impacts in the U.S. Since 1886, over 160 TCs have passed within 500 km of the North Carolina coast, with a TC crossing the state every 2–3 years^8^. Additionally, the coastal plains of ENC have a limited elevation range, enhancing its vulnerability to prolonged flooding^9^. Since 1999, Hurricanes Floyd, Matthew, and Florence set record rainfall and discharges in ENC river basins^4,10^ (Fig. 1), resulting in historic flooding.

Global mean TC wind speeds, precipitation rates, and intensity are projected to increase by the late twenty-first century^7,11,12^, which underscores the need to examine how the most devastating storms in ENC may be enhanced by climate change. Recent extreme rainfall events illustrate that rainfall probability statistics based on stationarity assumptions can no longer represent the rapidly changing climate^10,13,14^.

To explore changes in extreme rainfall at ‘2100’, the Design-Rainfall Approach (DRA) is proposed, where observed rainfall statistics are adjusted for changes in probability statistics derived from the dynamically downscaled global climate models (GCMs). Here, we do not explicitly model future hurricanes; rather, we examine potential changes in storm total precipitation informed by projections of future climatic conditions. The downscaled projections reflect the presence of TCs, but their strength is underestimated due to the resolution of the projections^15–17^. The influences of model resolution and statistical stationarity are mitigated with DRA by applying the probability statistics to two dynamically downscaled future periods. Scenarios are used from two GCMs: Community Earth System Model version 1 (a.k.a., the fourth version of the Community Climate System Model–CCSM4) (CESM)^18^ and Geophysical Fluid Dynamics Laboratory Coupled Model (CM3)^19^. Both models are members of the Coupled Model Intercomparison Project Phase 5 (CMIP5) ensemble^20^. The GCMs are dynamically downscaled to a 36-km horizontal grid spacing over the contiguous U.S. using the Weather Research and Forecasting (WRF) model^21^ under two Representative Concentration Pathways (RCPs)^22^ to create three scenarios: RCP4.5^23^ (CESM_4.5) and RCP8.5^24^ (CESM_8.5 and CM3_8.5). None of the data sets were bias-corrected; the individual model biases are deemphasized because DRA compares the relative changes in the projections between two future periods. Using the DRA is proposed for quantifying potential changes in extreme precipitation, which can be a prelude to quantifying flooding impacts from that precipitation.

## RESULTS

### Observational data

*Hurricane Floyd* made landfall near Cape Fear, NC, on 16 September 1999 as a Category 2 hurricane^25^, with a maximum storm surge of 3.1 meters. The recorded 4-day maximum storm intensity (MI) at Southport, NC, was 611 mm^26^. Floyd ranks 9th in a nationwide analysis of the highest 4-day TC rainfall observed during 1949–2018^4^. *Hurricane Matthew* made landfall near McClellanville, SC, on 8 October 2016 as a Category 1 hurricane^25^, with a storm surge of 1.8 meters at Hatteras, NC. The 3-day MI of 481 mm was measured near Evergreen, NC^26^. The combination of storm surge inundation and inland freshwater flooding from excessive rainfall from Matthew deluged ENC, forcing closure of two major interstates. Matthew—a 3-day storm for ENC—ranks 23rd in 4-day TC rainfall^4^. Hurricane Florence made landfall near Wrightsville Beach, NC, on 14 September 2018 as a Category 1 hurricane^25^. The maximum storm surge from Florence was recorded in New Bern, NC at 3.2 m. The rainfall associated with Florence shattered the state record with a 4-day MI of 913 mm near Elizabethtown, NC^26^, surpassing the previous record set by Floyd. Florence ranks 3rd in 4-day TC rainfall^4^. The widespread inland rainfall and storm surge from Florence triggered unprecedented flooding, contributing to economic losses that exceeded those from Floyd and Matthew combined^27^.

**Fig. 2:**
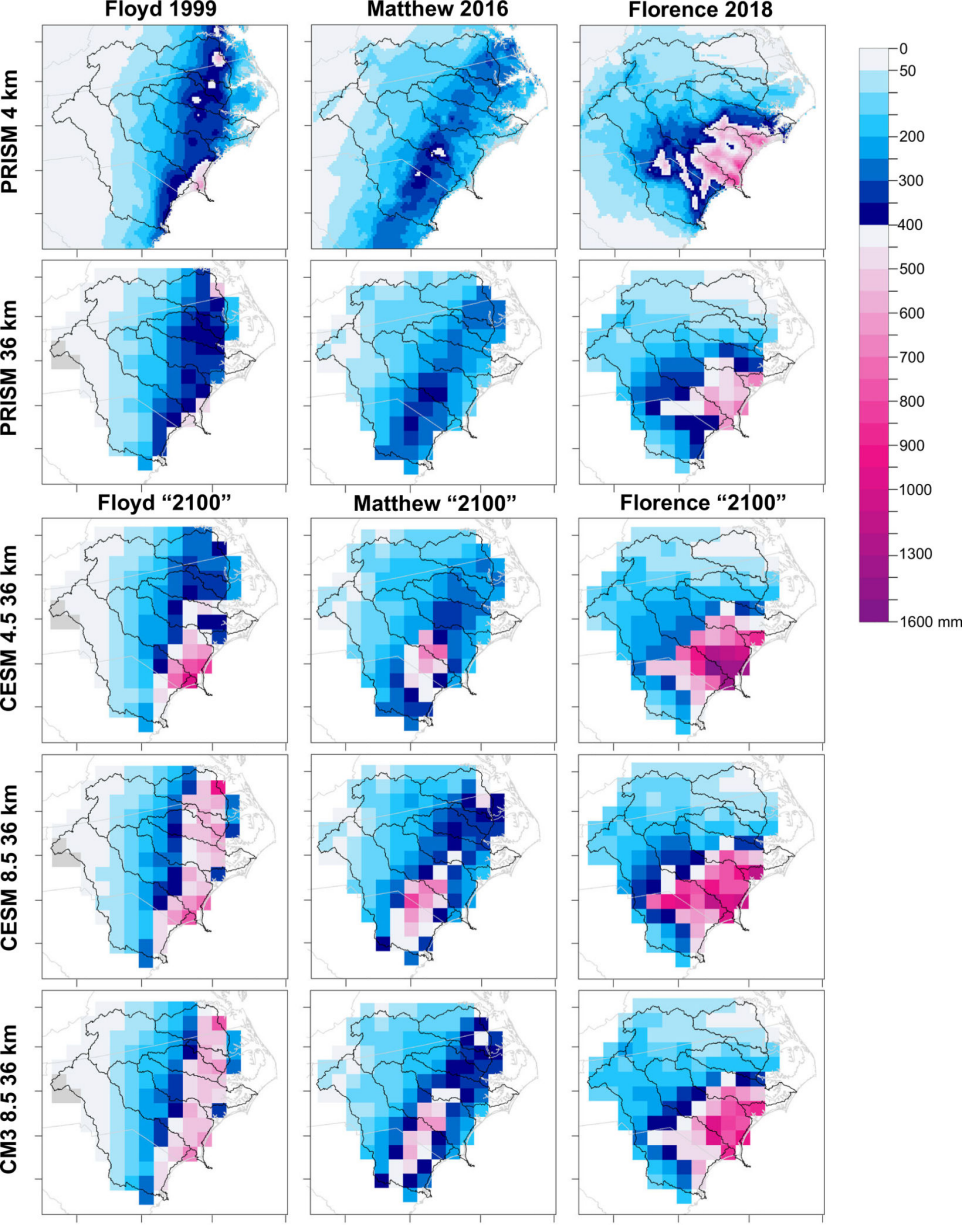
Tropical cyclone (TC) rainfall from PRISM4, PRISM36, and three ‘2100’ scenarios. The area shown in each panel is the 121-cell subset of the WRF domain representing the study area given in Fig. 1. The change from blue to pink in the color scale occurs at 400 mm, which corresponds to the mean 1000-yr rainfall in the study area (386 mm for 3-day duration and 397 mm for 4-day duration) from NOAA Atlas 14^29^.

Daily gridded precipitation data from the Parameter-elevation Regressions on Independent Slopes Model (PRISM)^28^ are used to characterize the observed conditions for two storm durations (3-day and 4-day rainfall) at 4-km grid spacing (PRISM4) for six ENC watersheds (Figs. 1 and 2). The observed MI in Floyd was 611 mm^26^, which is comparable to PRISM4 at 610 mm. For Matthew, the MI is underestimated in PRISM4: 481 mm^26^ vs. 460 mm. In Florence, the observed MI of 913 mm^26^ is slightly overestimated in PRISM4 with 917 mm. Overall, MI differences are <5%, concluding that PRISM4 faithfully represented the three events, so other observation-based, gridded data sets were not explored.

To facilitate comparison with the three WRF scenarios, PRISM4 was aggregated to the subset of the 36-km WRF domain (PRISM36) comprised of 121 grid cells, which reduced the MI by 17% for Floyd, 18% for Matthew, and 26% for Florence (Fig. 2), and underestimated the average storm intensity (AI) by 2% in Floyd 5% in Matthew, and 7% in Florence (Fig. 2). The regridding increased total rainfall (TR) by 14% in Floyd, 13% in Matthew, and 12% in Florence (Fig. 2) because the spatial coverage of ENC increases as the 36-km cells extend beyond the watershed borders (Fig. 2).

For many governmental agencies that manage stormwater in the U.S., the NOAA Atlas 14^29^ (Atlas 14) serves as the primary reference to acquire the probability that a precipitation event of a specific duration and intensity will occur during some period of years (i.e., return period, RP) at a given geographic location. For each TC at its observed duration, every PRISM36 cell was assigned the precipitation frequency estimate (RP) corresponding to rainfall occurring within the RP’s confidence intervals (CIs) provided by Atlas 14 precipitation frequency data^29^. In PRISM36, the rainfall from Floyd was a 100-yr or lower RP event for 83% of the cells, a 200-yr event for 7% of the cells, a 500-yr event in 8 cells, and exceeded the CIs for 1000-yr event in one cell (Fig. 2 and Table 1). Matthew in PRISM36, classified 93% of rainfall from a 100-yr or lower RP event, while 7% of the grid cells received 200 and 500-yr rainfall (Fig. 2). More severely, 26% of PRISM36 in ENC received a 200-yr or rarer event with Florence: 5 cells received 200-yr events, 10 grid cells received 500-yr events, 12 cells received 1000-yr events, and 5 cells received precipitation that exceeded the CI for a 1000-yr event (Fig. 2). Based on Atlas 14^29^ and at this scale, Florence was a 1000-yr or rarer event for more than 22,000 km^2^. In PRISM4, the local intensities are much more dramatic (Fig. 2).

### Future scenarios

Rainfall intensities for each storm scenario at the end of the twenty-first century—the ‘2100’ storm—were derived using DRA by applying the changes in projected precipitation frequency between two 30-year future periods: 2025–2054 (P_1_) and 2070–2099 (P_2_) to the observed rainfall from the TCs (PRISM36) using each cell’s RP. CIs derived for RPs during P_1_ in the three scenarios were within ±10% of the 2-, 5-, 10-, 25-, and 50- yr RPs. In the 100-, 200- and 500-yr RPs, the 10% CI did not exceed 10%, and the 90% CI increased up to 20% (Supplementary Figs. 1 and 2). The 1000-year RP CI envelope increased in 10% CI by up to 20% and 90% CI increased by up to 30%. In P_2_, the CIs in the 2-, 5- and 10-yr RPs are within ±10%; in rarer RPs, the 90% CI increases by up to 50% for a 1000-yr event, while the 10% RP increases only up to 20% (Supplementary Figs. 1 and 2).

**Fig. 3:**
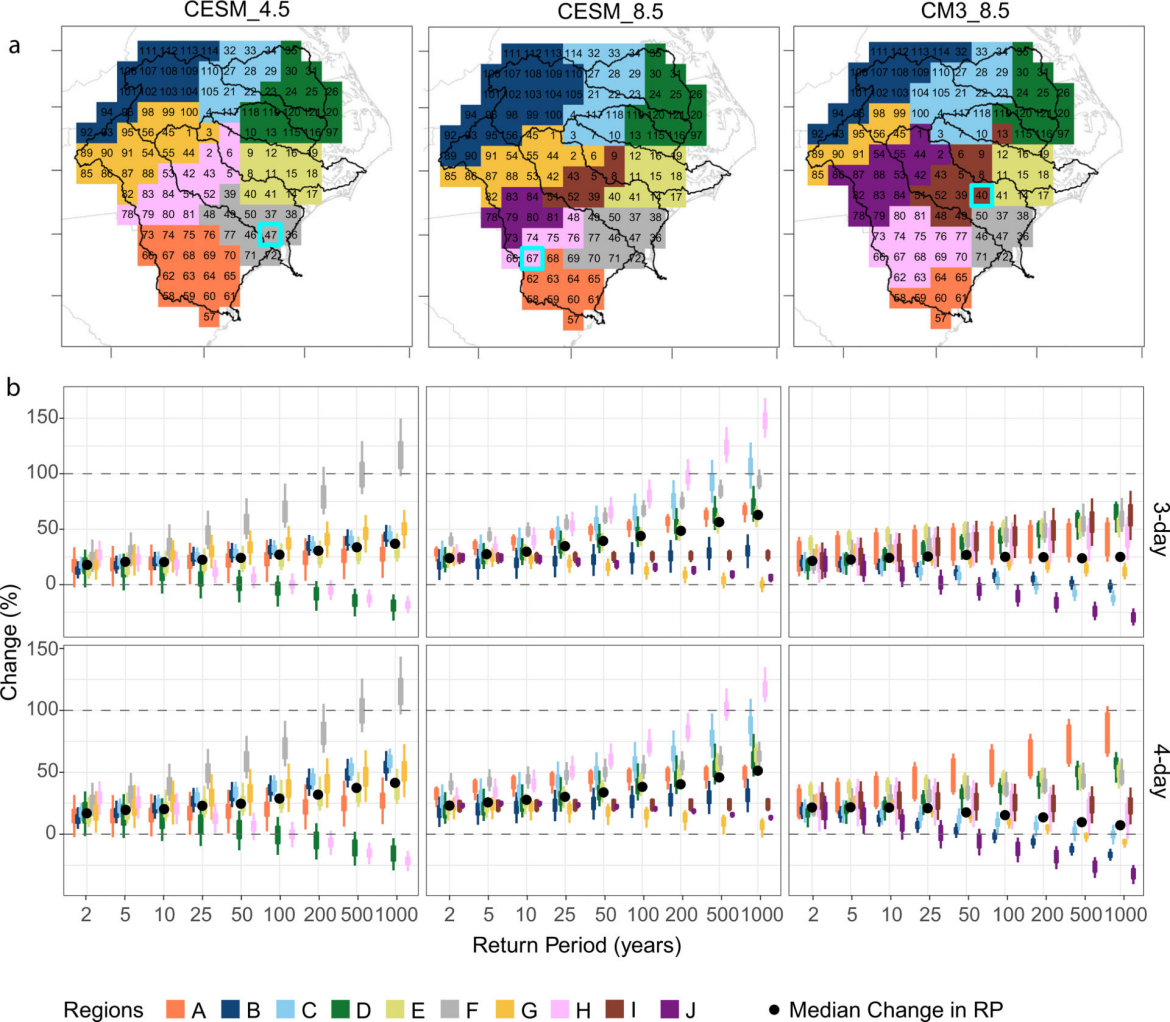
Localized changes in the return periods for 3-day and 4-day precipitation across ENC at ‘2100’ under three scenarios. **a** ENC river basins (see Fig. 1) with sub-regions (A–J) derived from regional frequency analysis (RFA) for each scenario. Numbers are indexed from 1 to 121 for the 36-km WRF grid cells in ENC. Teal boxes mark grid cells with maximum change. **b** Change (%) at ‘2100’ in every RFA sub-region by scenario in columns (left-to-right: CESM_4.5, CESM_8.5, and CM3_8.5), and by storm duration in rows (top-to-bottom: 3-day and 4-day durations). Black dots represent the median change in each RP. Dashed lines are indicated at 0% (no change) and 100%.

ENC TR increases at ‘2100’ under CESM_4.5 by 25% in Floyd, 22% in Matthew, and 41% in Florence (Fig. 2 and Table 1). Increases in MI are substantially higher: 954 mm (+89%) in Floyd, 714 mm (+90%) in Matthew, and 1535 mm (+130%) in Florence (Fig. 2). The largest increases in 100-yr and higher frequencies in both durations were modeled in the Lower Cape Fear River Basin (region A in Fig. 3). CESM_4.5 simulated a 20% decrease in the 100-yr and longer RPs in central ENC, but ~30% increase closer to the coast, with the maximum increase of 150% recorded in the 1000-yr RP in grid cell 47 (Fig. 3). In CESM_4.5, RPs of <100 years increased by ~20% (Fig. 3). The area of ENC receiving precipitation exceeding the CI of a 1000-yr storm could increase by a factor of seven in Floyd, by a factor of five in Florence, and increase from 0 to 7 cells in Matthew (Fig. 2).

Changes in CESM_8.5 are more dramatic than in CESM_4.5, with TR increasing by 43% (Floyd), 42% (Matthew), and 44% (Florence) (Fig. 2, Table 1). Changes in future extreme rainfall in CESM_8.5 were more intense but distributed differently than in CESM_4.5, resulting in an overall increase but lower MI than in CESM_4.5: 913 mm (+81%) in Floyd, 819 mm (+118%) in Matthew, and 1141 mm (+71%) in Florence (Fig. 2). The largest increases in 100-yr and longer RPs for both durations were modeled in the lower Pee-Dee Basin (region A in Fig. 3). CESM_8.5 projected a 0 to 10% decrease in the 100-yr and larger RP intensities in the upper Pee-Dee and Neuse Rivers, and an average of 50% increase elsewhere in the study area. The maximum increase recorded was 168% in the 1000-yr RP in 3-day duration in grid cell 67 (Fig. 3). In CESM_8.5, RPs of <100 years increased ~33% (Fig. 3). The area of ENC with precipitation exceeding CI of a 1000-yr storm increased by factors of 16, 8, and 6 in Floyd, Matthew, and Florence, respectively (Fig. 2).

Like CESM_8.5, CM3_8.5 increases TR across ENC at ‘2100’ by 34% in Floyd, 34% in Matthew, and 29% in Florence (Fig. 2 and Table 1). Increases in MI in CM3_8.5 are substantial but lower than in both CESM simulations: 800 mm (+59%) in Floyd, 633 mm (+69%) in Matthew, and 977 mm (+46%) in Florence (Fig. 2 and Table 1). CM3_8.5 modeled ~30% increase in rainfall in the ENC coastal plains, but up to a 20% decrease in 100-year and larger RP intensities farther inland in ENC. The maximum increase within a grid cell was 85% in the 1000-yr RP in the lower reaches of the Pee-Dee Basin (grid cell 40; Fig. 3). In CM3_8.5, RPs of less than 100 years also increased by an average of 30% (Fig. 3). The area of ENC with precipitation exceeding CI of a 1000-yr storm grew by factors of eleven, eight, and five in Floyd, Matthew, and Florence, respectively, suggesting widespread torrential rainfall (Fig. 2).

## DISCUSSION

This study quantifies projected increases in extreme precipitation associated with three devastating TCs under three scenarios using the DRA methodology while acknowledging sources of uncertainty in the approach and scenarios. The DRA is based on the RPs assigned to each cell using the current Atlas 14^29^ for Virginia, North Carolina and South Carolina (published in 2006^29^), which omits over a dozen TCs including Matthew and Florence. Updating the Atlas 14^29^ rainfall statistics to include those events would likely reduce RPs and change CIs used here. Additionally, DRA uses 100-yr to 1000-yr rainfall frequencies based on 30-yr data series, which generates larger 90% CIs that may underestimate calculated intensities^30^. The modeled data are handicapped by coarse horizontal resolution for TCs and legacy biases from GCMs^15–17,30,31^, so the study was designed to explore relative changes in future rainfall independent of model biases^30^. Accordingly, the changes associated with aggregating the data or with GCM biases, factors that would likely underestimate the future precipitation maxima, and the variability within each cell were not corrected.

**Fig. 4:**
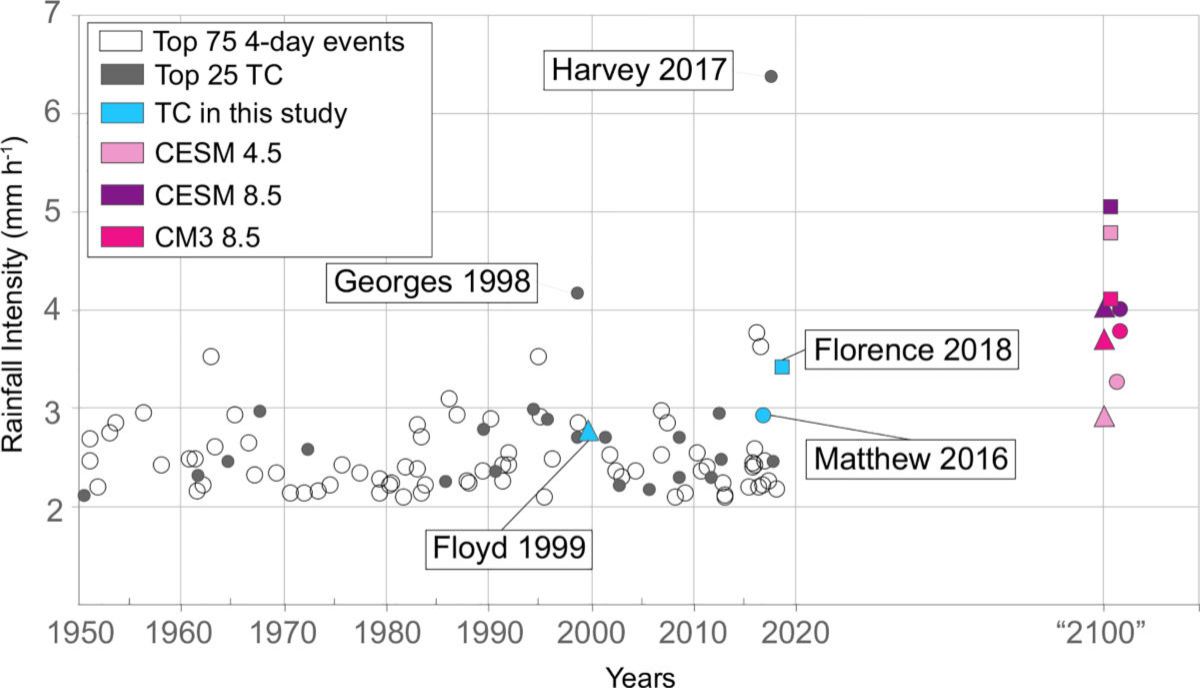
Rainfall intensity (mm h^−1^) of the top 100 4-day events in the U.S. from 1949 to 2018 and of projections at ‘2100’ using DRA. Rainfall intensity is based on the area of 2° × 2° (50,000 km^2^) area used in Kunkel & Champion^4^. The top 25 TCs (through 2018) are in filled circles, while the remaining top 75 4-day events are in open circles. Observed rainfall intensities from Floyd (triangle), Matthew (circle), and Florence (square) are highlighted in blue. Projected rainfall intensities at ‘2100’ from CESM_4.5, CESM_8.5, and CM3_8.5 are in pink, purple, and magenta, respectively, using subset^4^ of the projections for areas shown in Fig. 1. Four of the top 25 TCs made landfall in the study area, also including Gloria (1985)^4^, which was not examined here.

**Fig. 5:**
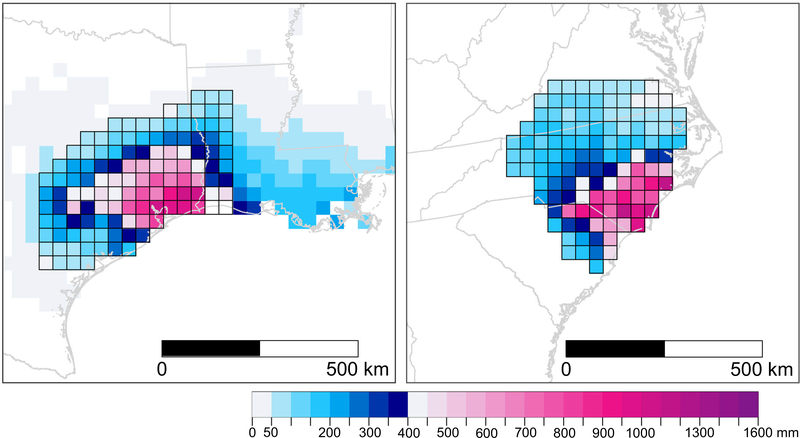
Observed Harvey and modeled Florence comparison. Comparison of the total rainfall (TR) from Harvey 2017 over 4 days from PRISM36 and Florence 2100 under CESM_8.5. TR for Harvey within the area corresponding to ENC (indicated in black grid net) was 53,808 Mt, and from Florence 2100 under CESM_8.5 was 47,809 Mt.

Floyd ‘2100’ from CESM_4.5 and CM3_8.5 have comparable TR to Florence 2018 in ENC (Table 1), suggesting that despite the conservative estimates of the extremes associated with the coarseness of the WRF model’s resolution, the DRA generated extreme rainfall totals similar to Florence 2018 as Floyd ‘2100’. Furthermore, Floyd ‘2100’ from CESM_4.5 and Matthew 2016 have similar AIs (Table 1). Projected increases in rainfall intensities may appear alarming, but the ‘2100’ values are reasonable in the context of extreme events in the U.S. in the last two decades (Fig. 4). The MI for Florence ‘2100’ under CESM_4.5 is 1535 mm, which is comparable to observed point rainfall during Harvey (1539 mm) in 2017 (Fig. 5). Additionally, AIs of the ‘2100’ TCs fall within the top 100 AIs reported in the U.S., and Florence ‘2100’ would rank in the top 4 events of 4-day duration^4^ (Fig. 4 and Table 1). Applying DRA to these scenarios suggests that the future projected rainfall for ENC is already underway. However, it is unclear whether precipitation intensities from Harvey (e.g., as Florence ‘2100’, Fig. 5) would be plausible from a tropical cyclone in NC, and additional research that incorporates potential changes to sea-surface temperature and other physical mechanisms would also be required. Yet, although this paper does not address the formation of the future hurricanes, precipitation from some of the ‘largest’ historical events have already been surpassed in NC; for example, the point rainfall maximum observed from Florence 2018 in NC was more than twice that from Katrina (2005) in the Gulf states.

Projected changes within each scenario are consistent for 3- and 4-day durations (Fig. 3 and Supplementary Figs. 1 and 2), but the spatial distribution varies between the sub-regions (Fig. 3). CESM_8.5 (regions B, G, I, J) and CM3_8.5 (regions B, C, G, J) project small or negative changes in rainfall intensities of 50 years and longer RPs in inland ENC, suggesting that the coastal plain of ENC may disproportionally incur increases in extreme TC rainfall. Projected increases along the coastal plain are more alarming (regions A, E, F, and H), as this area (except region H in CESM_8.5) is also susceptible to storm surge (which would be amplified by sea-level rise) and compound flooding, facilitated by low elevation.

Exploring changes in extreme precipitation by applying DRA to regional climate simulations quantifies potential future extremes including RPs beyond 200 years. Average increases based on statistical downscaling are more modest than those here: 20-yr daily precipitation at ‘2100’ under RCP4.5 and RCP8.5 is projected to increase by 13% and 21%, respectively^3^. Likewise, statistically downscaled projections of 25-, 50-, and 100-yr, 1- to 7-day duration precipitation increase by 10% and 20% for those RCPs^32^, while this study projects increases of 22% and 35%, respectively. Additionally, this study illustrates the potential for increases associated with these devastating storms of up to 44% in total storm precipitation and up to 130% in maximum storm intensity in 500-yr, 1000-yr, and longer RPs. The average increases of 24% (20%) in 3-day (4-day) duration under CM3_8.5 are comparable to those from ensemble averages from pseudo–global warming (PGW) experiments: 24%^33^ and 23%^34^. However, the average change in CESM_8.5 of 39% (36%) in 3-day (4-day) duration surpasses the PGW average. The study also shows that average increases in 3-day precipitation (24% in CM3_8.5 and 39% in CESM_8.5) are higher than increases in 4-day precipitation (20% in CM3_8.5 and 36% in CESM_8.5), while CESM_4.5 produced lower average changes in 3-day precipitation (24%) than in 4-day precipitation (25%). These differences between the scenarios and durations are not intuitively explained, so further investigation would be recommended in a future study. In addition to duration, there is a notable variation between RFA regions (Fig. 3). Since RFA regions were guided by the NCEI U.S. Climate Divisions, the variation in future changes could be an artifact of the coarse (36-km) horizontal grid spacing of the WRF data used in the study, which does not offer a gradual change between regions. Additional research with finer-resolution downscaled data is planned to explore whether decreasing the horizontal grid spacing (as in Jalowska & Spero^31^) would enhance the use of DRA.

The potential changes in TC extreme rainfall could portend devastating consequences for ENC. Topographical homogeneity in the coastal plain exacerbates ENC’s vulnerability^9^. DRA could be applied as a first step to quantify potential changes in pluvial and fluvial flooding associated with TCs and other extreme rainfall events (e.g., atmospheric rivers or convective storms) using other future data sets and geographic regions; extreme precipitation from snowfall was not examined here. This study neglected factors that would amplify impacts from TCs extreme rainfall, including wind speed, storm surge, compound flooding, and sea-level rise. Yet, the parallels between Floyd ‘2100’ and Matthew 2016 and Florence 2018 in ENC, as well as Florence ‘2100’ and Harvey 2017, corroborate emerging literature^35^. Regional planners in low-lying communities could benefit from using DRA in their resilience and adaptation strategies.

## METHODS

### Observational data

TC dates were obtained from NOAA (https://www.wpc.ncep.noaa.gov/tropical/rain/tcname.html): 14–17 September 1999 (Floyd), 7–9 October 2016 (Matthew), and 14–17 September 2018 (Florence). PRISM rainfall (http://www.prism.oregonstate.edu) for each TC was summed over its duration, and rainfall totals were validated against NOAA data sets. Distance-average weighted remapping was used—increasing the neighboring cells from 4 to 80—to transform PRISM4 to PRISM36 using 9 × 9 cells with REMAPDIS function from Climate Data Operators (https://code.mpimet.mpg.de/projects/cdo/). The PRISM36 was then subset for 121 WRF cells within ENC watersheds (Figs. 1 and 3). Using the latitude and longitude associated with each WRF cell, total rainfall in each cell per TC was allocated to the corresponding annual maximum series (AMS) rainfall frequency estimates (RPs) with 10% and 90% CIs from NOAA Atlas 14 precipitation frequency data^29^ (https://hdsc.nws.noaa.gov/hdsc/pfds/pfds_map_cont.html). Although Florence rained over ENC for 5 days, maximum 4-day rainfall was used to align with the maximum period available in Atlas 14^29^. The cells with recorded rainfall exceeding 90% CI of a 1000-yr rainfall in Atlas 14^29^ were assigned RP of a 1000-yr rainfall event.

### Future scenarios

Two members of the Coupled Model Intercomparison Project Phase 5 (CMIP5) ensemble^20^—the Community Earth System Model version 1 (a.k.a., the fourth version of the Community Climate System Model—CCCM4) (CESM)^18^ (RCP4.5^23^ and RCP8.5^24^) and Geophysical Fluid Dynamics Laboratory Coupled Model (CM3)^19^ (RCP8.5^24^)—were dynamically downscaled^36,37^ for this study. These models were chosen based on internal commitments and stakeholder needs. Neither CM3 nor CESM is an outlier^20^ in the CMIP5 ensemble. Both underestimate the trends in temperature change in historical data but reproduce the mean temperatures very well over the CONUS^38^. Although CESM has a finer native resolution than CM3 (Supplementary Fig. 3 and Supplementary Table 1), both models produced average biases of the mean precipitation values, mostly overestimating the mean precipitation compared with other CMIP5 members^20^. Neither the GCMs nor the downscaled projections were bias-corrected for this study.

### Dynamical downscaling

Dynamical downscaling is computationally intensive, which inherently limits the number of models and scenarios that can be processed. The three selected GCM scenarios were dynamically downscaled to 36-km horizontal grid spacing over the contiguous U.S. using the Weather Research and Forecasting model (WRF)^21^ with the options shown in Supplementary Table 1. These downscaling techniques were vetted against historical cases that emulated future climate downscaling studies^36,37,39–43^ to lend confidence in their viability for this work. The dynamically downscaled data sets for future climate scenarios are part of a suite of internally developed data with common geographic domains and grid structures, which promoted consistency in this study. These downscaled data were used in analyses shown in high-level National documents^44,45^. A preliminary analysis of the downscaled projections for 2025–2035 is presented by Nolte et al.^46^ Hourly rainfall from WRF was archived for 2025–2100 for each scenario and, for this study, subset for 121 grid cells within six ENC watersheds (Figs. 1 and 3).

### Probability rainfall statistics

Precipitation intensity-duration-frequency (PIDF) curves represent the probability that precipitation of a given intensity and duration will occur at a location. Intensity refers to an amount of precipitation falling per unit time, duration refers to a time unit with continuous precipitation at that intensity, and frequency (i.e., RP) indicates the probability of occurrence for a storm of a given duration and intensity^47,48^. PIDF curves require at least 20 years of hourly precipitation data^29,49^.

Modeled precipitation duration used hourly rainfall with a minimum accumulation of 1 mm hr^−1 31^ with a rolling summation for 3- and 4-day durations. The highest annual rainfall accumulation for each duration was used to develop AMS. The AMS was fit with generalized extreme value (GEV) distribution using regional frequency analysis (RFA) based on L-moments^50^ using R package lmomRFA^51^. The RFA tailors probability distributions to a region composed of different sites characterized by similar event frequencies. Each modeled grid cell was treated as an individual site for RFA. Guided by NCEI U.S. Climate Divisions (https://psl.noaa.gov/data/usclimdivs/data/map.html), cells were grouped into RFA regions and tested for homogeneity and discordancy (Fig. 3 and Supplementary Tables 2, 3). The AMS was subset into two 30-year periods P_1_ (2025–2054) and P_2_ (2070–2099). L-moments were computed for each cell in each scenario for both durations in P_1_ and P_2_. The GEV frequency distribution was fitted to a vector of regional average L-moments, computing estimated quantiles from “regional frequency distribution” with 10% and 90% CIs (using 1000 simulations) for RPs of 2, 5, 10, 25, 50, 100, 200, 500, and 1000 years^51^. The procedure was repeated for each scenario and duration.

### Design-rainfall approach (DRA)

PIDF curves can be used to derive a design-rainfall that is further used to develop a design flood required to be considered in the design of managed stormwater, e.g., using drainage systems or dams. The design-rainfall concept is to compose a synthetic storm by adjusting the observed storm precipitation intensities to return periods of choice.

In this study, a Design-Rainfall Approach (DRA) was introduced, where observed storm RPs were adjusted by percent change derived from future PIDF curves. Future PIDF curves were developed for each WRF cell for P_1_ and P_2_ under each scenario. Relative changes in precipitation between P_1_ and P_2_ were used to calculate the extreme rainfall at ‘2100’. The changes were applied to PRISM36 precipitation using the corresponding RPs assigned to each ENC cell from Atlas 14^29^. The projected precipitation in each cell was then assigned an updated RP (based on a current RP from Atlas 14^29^) to analyze the spatial distribution of future changes in precipitation associated with these storms (Fig. 2).

## Supplementary Material

Supplement1

## Figures and Tables

**Table 1.** Change in rainfall.

Scenario	Tropical cyclone	TR (Mt)	% Change in TR	MI (mm)	% Change in MI	AI (mm hr ^−1^)

PRISM36 (regridded historical)	Floyd	26,064		504		2.8
	Matthew	26,876		375		2.9
	Florence	33,109		667		3.4
CESM_4.5 '2100'	Floyd	32,597	25%	954	89%	2.9
	Matthew	32,891	22%	714	90%	3.2
	Florence	46,797	41%	1535	130%	4.8
CESM_8.5 '2100'	Floyd	37,169	43%	913	81%	4.0
	Matthew	38,284	42%	819	118%	4.0
	Florence	47,809	44%	1141	71%	5.1
CM3_8.5 '2100'	Floyd	34,906	34%	800	59%	3.7
	Matthew	36,127	34%	633	69%	3.8
	Florence	42,585	29%	977	46%	4.1

Change in the total rainfall (TR) in the study area, in megatons (Mt), and in maximum intensity (MI) in mm. Average intensity (AI) was calculated for each event using the area^4^ in Fig. 1.
