# Supplementary material for: Projecting changes in extreme rainfall from three tropical cyclones using the design-rainfall approach
Source: Nat Clim Chang. Author manuscript; Available in PMC 2022 Mar 25. (PMC8128695)
Supplement: Supplement1 [file NIHMS1694690-supplement-Supplement1.docx]

Supplementary Figures


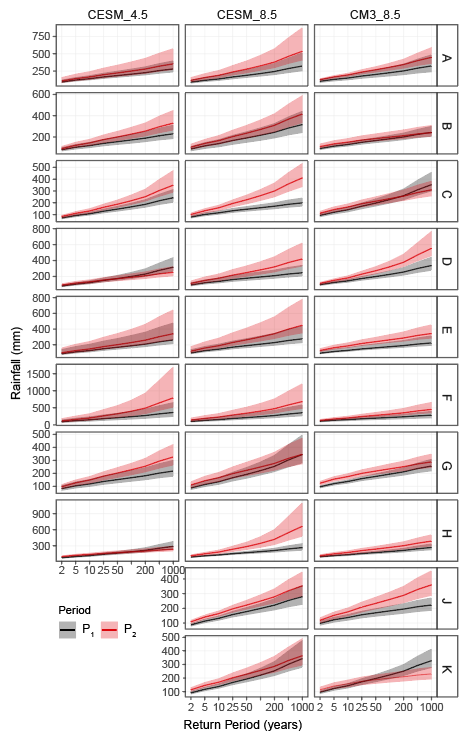


Supplementary Figure 1: PIDF curves for P_1_ (gray) and P_2_ (red) by sub-region (Fig. 3a) for 3-day duration. Median PIDF curves are highlighted in darker lines. The ribbons correspond to lowest 10% confidence interval (CI) and highest 90% CI within cells in the regions. Note that the scales on the y-axis vary by region, as do the specific areas represented by each region for each scenario.


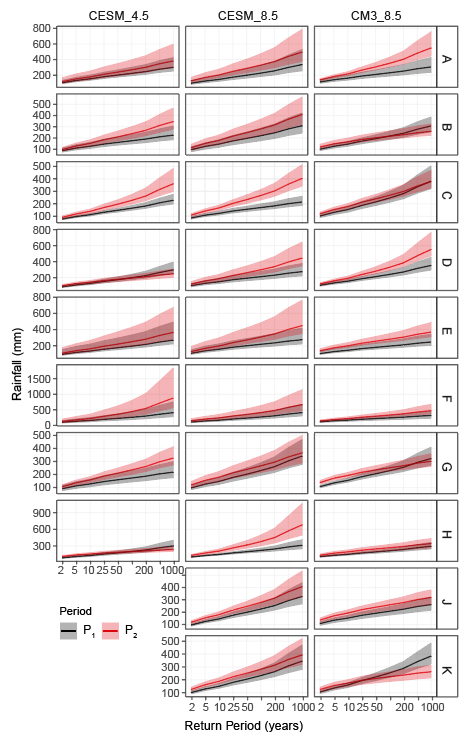


Supplementary Figure 2: Same as Supplementary Figure 1, but for 4-day duration.

| **a** CM3 (2° x 2.5°) | **b** CESM (0.875° x 1.25°) |
| --- | --- |
| 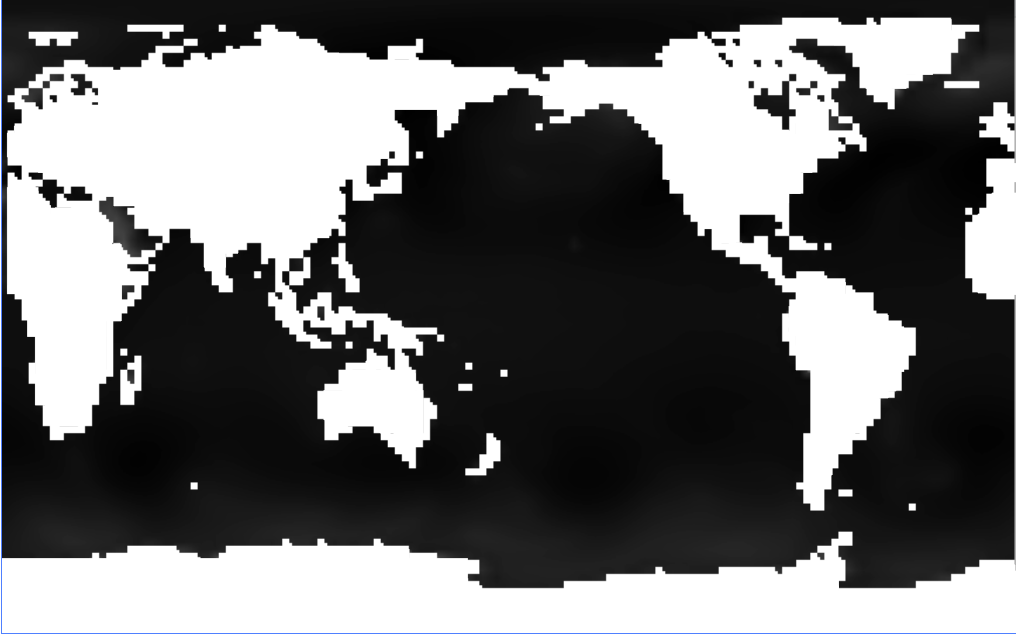 | 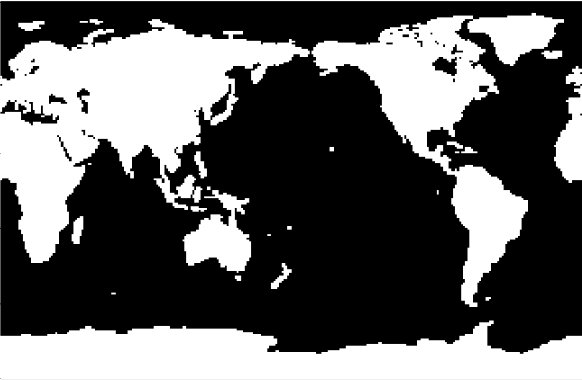 |
| **c** WRF-CM3 (108 km) |  |
| 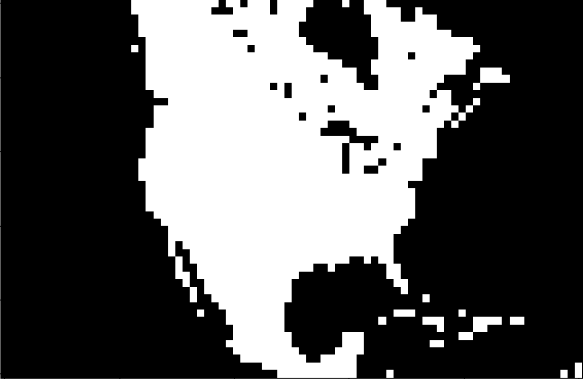 |  |
| **d** WRF -CM3 and WRF-CESM (36 km) | |
| 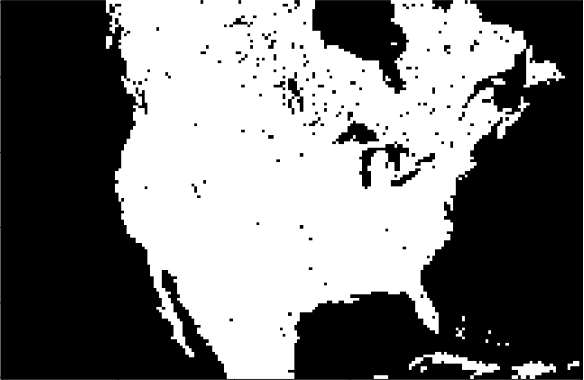 | |

Supplementary Figure 3. Geographic domains of the models used in the study. a Global projection for CM3 at its native resolution. b Global projection for CESM at its native resolution. c WRF intermediate downscaling of CM3 to 108-km horizontal grid spacing as part of two-way nesting (not used in the analysis). d WRF domain in Lambert conformal projection with 36-km horizontal grid spacing used for both GCMs.

Supplementary Tables

Supplementary Table 1. WRF model configurations used for dynamically downscaling the GCMs.

|  | CM3 | CESM |
| --- | --- | --- |
| GCM Resolution | 2° x 2.5° | 0.875° x 1.25° |
| WRF Domain | 108-36-km, two-way nest | 36-km only |
| WRF Version | 3.6 | 3.4.1 |
| Scenarios^A^ | RCP8.5 | RCP4.5 and RCP8.5 |
| Radiative Forcing^B^ | Follows RCPs | Standard |
| Lake Temperatures^C^ | Modeled with FLake following^1^ | Imported from the Community Land Model following^2^ |
| Shortwave Radiation | RRTMG | RRTMG |
| Longwave Radiation | RRTMG | RRTMG |
| Microphysics | WSM6 | WSM6 |
| Convective Parameterization Scheme^D^ | Kain-Fritsch with radiative feedback following^3^ | Kain-Fritsch with radiative feedback following^3^ |
| Planetary Boundary Layer | YSU | YSU |
| Land-Surface Model | Noah | Noah |
| Nudging | Spectral nudging above PBL toward GCM^4^ | Spectral nudging above PBL toward GCM^4^ |
| Land use | 24-category USGS | 24-category USGS |

^A^ RCP4.5 with CM3 was not downscaled as part of this suite of data, so those data were not available to be used in this study.

^B^ The option to use radiative forcing that follows the RCPs was added to WRFv3.5, so it was not available for the downscaling of CESM using WRFv3.4.1.

^C^ Spero et al. (2016) demonstrated the need for better defining subgrid lake temperatures for dynamical downscaling. In general, lake temperature data were not available with the CMIP5 archive for downscaling. Here, CESM was downscaled with WRF using the technique described in Spero et al. (2016). The analogous data for CM3 were not available in the CMIP5 archive, so the explicit lake model, FLake, was used to downscale CM3 with WRF following Mallard et al. (2014).

^D^ Both CM3 and CESM were downscaled using the Kain-Fritsch convective parameterization scheme with radiative feedback following Herwehe et al. (2014). Those changes were publicly released in WRFv3.6, which was used “as is” to downscale CM3. To downscale CESM, those changes were implemented into WRFv3.4.1 and used as a pre-release code. The versions of the convective scheme are otherwise the same, although all science updates and bug fixes that were incorporated into WRF between v3.4.1 and v3.6 were included in the CM3 downscaling.

Supplementary Table 2. Results of heterogeneity tests, H(i), in RFA GEV distribution fitting. H(i) was calculated using the *regtst* function from R package^5^. Regions are indicated in Fig. 3a.

|  | CESM_4.5 | | CESM_8.5 | | CM3_8.5 | |
| --- | --- | --- | --- | --- | --- | --- |
| region | H(i)  3-day | H(i)  4-day | H(i)  3-day | H(i)  4-day | H(i)  3-day | H(i)  4-day |
| A | -0.2 | -0.4 | -1.4 | -1.6 | -2 | -1.9 |
| B | -1.2 | -1 | -1.7 | -1.9 | 1.5 | -0.5 |
| C | -1.4 | -2 | -1.2 | -1.7 | -0.4 | -0.5 |
| D | -0.7 | 0.4 | 1 | 1 | -1.5 | -1.2 |
| E | -1 | -0.1 | -1.6 | -1.7 | -1.8 | -1.9 |
| F | 1.4 | 1.4 | -1.5 | -1.8 | -1.9 | -1.9 |
| G | 0.2 | 0.2 | -1.1 | -1.1 | -1.4 | -1.8 |
| H | -1.8 | -1.6 | -1.7 | -1.7 | -1.4 | -1.3 |
| J | N/A | N/A | -1.6 | -1.9 | -0.7 | -0.5 |
| K | N/A | N/A | -1.3 | -1 | -1.1 | -0.9 |

Supplementary Table 3. Results of discordancy tests, D(i), in RFA GEV distribution fitting. D(i) was calculated using *regtst* from R^5^. Regions and cell numbers are indicated in Fig. 3a.

| CESM_4.5 | | | | CESM_8.5 | | | | | CM3_8.5 | | | | |
| --- | --- | --- | --- | --- | --- | --- | --- | --- | --- | --- | --- | --- | --- |
| Region | Cell number | D(i) 3-day | D(i) 4-day | Region | Cell number | D(i) 3-day | D(i) 4-day | Region | | Cell number | D(i) 3-day | D(i) 4-day |  |
| G | 1 | 0.15 | 0.09 | G | 1 | 1.4 | 1.4 | K | | 1 | 0.7 | 0.9 |  |
| H | 2 | 0.04 | 0.41 | G | 2 | 0.4 | 0.1 | K | | 2 | 2.2 | 2.0 |  |
| G | 3 | 0.17 | 0.03 | C | 3 | 0.8 | 1.0 | C | | 3 | 1.2 | 1.1 |  |
| C | 4 | 2.52 | 2.17 | C | 4 | 2.7 | 2.7 | C | | 4 | 0.6 | 0.6 |  |
| H | 5 | 0.66 | 0.26 | J | 5 | 1.8 | 0.9 | J | | 5 | 0.6 | 0.5 |  |
| H | 6 | 0.89 | 0.46 | G | 6 | 0.6 | 0.5 | J | | 6 | 1.3 | 0.8 |  |
| H | 7 | 0.67 | 0.68 | C | 7 | 0.4 | 0.5 | C | | 7 | 0.9 | 0.7 |  |
| E | 8 | 0.46 | 0.87 | J | 8 | 1.1 | 1.3 | J | | 8 | 1.7 | 1.4 |  |
| E | 9 | 0.99 | 1.20 | J | 9 | 1.1 | 1.1 | J | | 9 | 0.3 | 0.3 |  |
| D | 10 | 0.82 | 1.02 | C | 10 | 2.2 | 1.7 | C | | 10 | 0.6 | 1.7 |  |
| E | 11 | 0.32 | 0.05 | E | 11 | 0.3 | 0.4 | E | | 11 | 0.4 | 0.2 |  |
| E | 12 | 1.02 | 0.36 | E | 12 | 1.4 | 0.8 | E | | 12 | 1.9 | 1.8 |  |
| D | 13 | 0.28 | 0.46 | D | 13 | 1.8 | 0.8 | J | | 13 | 0.4 | 0.5 |  |
| E | 14 | 1.17 | 1.31 | E | 14 | 0.9 | 0.4 | E | | 14 | 0.8 | 0.3 |  |
| E | 15 | 1.05 | 0.38 | E | 15 | 0.5 | 1.4 | E | | 15 | 1.5 | 0.4 |  |
| E | 16 | 1.03 | 1.89 | E | 16 | 2.0 | 2.3 | E | | 16 | 1.0 | 2.2 |  |
| E | 17 | 0.67 | 0.98 | E | 17 | 1.7 | 2.2 | E | | 17 | 0.3 | 1.5 |  |
| E | 18 | 2.34 | 1.72 | E | 18 | 1.6 | 0.3 | E | | 18 | 0.9 | 0.3 |  |
| E | 19 | 0.47 | 0.46 | E | 19 | 1.1 | 0.7 | E | | 19 | 1.4 | 1.5 |  |
| D | 20 | 0.16 | 0.88 | D | 20 | 0.7 | 0.7 | D | | 20 | 0.4 | 0.4 |  |
| C | 21 | 2.06 | 1.93 | C | 21 | 0.0 | 0.1 | C | | 21 | 0.8 | 0.8 |  |
| C | 22 | 0.74 | 0.37 | C | 22 | 0.8 | 0.5 | C | | 22 | 0.5 | 1.5 |  |
| D | 23 | 0.60 | 0.79 | C | 23 | 0.5 | 0.8 | C | | 23 | 0.7 | 0.5 |  |
| D | 24 | 0.69 | 0.46 | D | 24 | 0.6 | 0.5 | D | | 24 | 1.2 | 0.8 |  |
| D | 25 | 0.68 | 1.19 | D | 25 | 0.5 | 0.2 | D | | 25 | 0.3 | 0.2 |  |
| D | 26 | 2.30 | 2.42 | D | 26 | 0.6 | 0.7 | D | | 26 | 0.6 | 0.8 |  |
| C | 27 | 0.07 | 0.02 | C | 27 | 0.7 | 1.1 | C | | 27 | 0.9 | 0.5 |  |
| C | 28 | 0.08 | 0.76 | C | 28 | 0.5 | 0.9 | C | | 28 | 2.0 | 1.8 |  |
| C | 29 | 0.36 | 0.45 | C | 29 | 0.9 | 0.9 | C | | 29 | 1.7 | 1.7 |  |
| D | 30 | 0.98 | 0.25 | D | 30 | 1.2 | 1.5 | D | | 30 | 1.0 | 1.0 |  |
| D | 31 | 1.91 | 1.23 | D | 31 | 1.1 | 1.3 | D | | 31 | 0.6 | 0.9 |  |
| C | 32 | 0.09 | 1.87 | C | 32 | 0.5 | 0.4 | B | | 32 | 1.8 | 0.9 |  |
| C | 33 | 0.49 | 0.44 | C | 33 | 0.2 | 0.5 | C | | 33 | 0.9 | 1.4 |  |
| C | 34 | 1.58 | 1.70 | C | 34 | 0.8 | 0.4 | C | | 34 | 1.4 | 1.2 |  |
| D | 35 | 1.81 | 1.39 | D | 35 | 1.6 | 1.9 | D | | 35 | 0.3 | 1.2 |  |
| F | 36 | 0.34 | 0.26 | F | 36 | 1.2 | 1.3 | F | | 36 | 1.2 | 1.1 |  |
| F | 37 | 0.59 | 0.31 | F | 37 | 0.5 | 0.7 | F | | 37 | 1.9 | 2.2 |  |
| F | 38 | 0.81 | 0.37 | F | 38 | 2.0 | 1.0 | F | | 38 | 0.6 | 1.5 |  |
| F | 39 | 1.47 | 1.10 | J | 39 | 0.8 | 1.6 | J | | 39 | 1.0 | 2.0 |  |
| E | 40 | 0.86 | 1.19 | E | 40 | 0.2 | 1.1 | J | | 40 | 0.9 | 0.7 |  |
| E | 41 | 1.63 | 1.59 | E | 41 | 0.3 | 0.3 | E | | 41 | 0.8 | 0.7 |  |
| H | 42 | 1.46 | 1.26 | G | 42 | 0.1 | 0.6 | K | | 42 | 0.9 | 1.2 |  |
| H | 43 | 0.74 | 0.70 | J | 43 | 0.4 | 0.2 | J | | 43 | 0.7 | 1.1 |  |
| G | 44 | 0.17 | 0.01 | G | 44 | 2.1 | 2.3 | K | | 44 | 1.6 | 0.6 |  |
| G | 45 | 0.84 | 0.71 | G | 45 | 0.8 | 0.5 | G | | 45 | 0.9 | 1.0 |  |
| F | 46 | 0.27 | 1.04 | F | 46 | 1.3 | 1.6 | F | | 46 | 0.4 | 0.5 |  |
| F | 47 | 1.13 | 1.03 | F | 47 | 1.7 | 1.7 | F | | 47 | 1.3 | 0.4 |  |
| F | 48 | 1.60 | 0.90 | H | 48 | 1.2 | 1.2 | J | | 48 | 2.4 | 2.0 |  |
| F | 49 | 2.33 | 2.06 | F | 49 | 0.7 | 1.1 | J | | 49 | 0.4 | 0.7 |  |
| F | 50 | 0.33 | 0.41 | F | 50 | 0.8 | 1.2 | F | | 50 | 0.4 | 0.7 |  |
| H | 51 | 2.10 | 0.78 | J | 51 | 1.1 | 0.5 | J | | 51 | 1.1 | 1.0 |  |
| H | 52 | 2.54 | 1.90 | J | 52 | 0.7 | 1.3 | J | | 52 | 1.2 | 0.9 |  |
| H | 53 | 0.56 | 0.79 | G | 53 | 0.3 | 0.3 | K | | 53 | 0.6 | 0.7 |  |
| G | 54 | 0.45 | 0.27 | G | 54 | 0.8 | 0.3 | K | | 54 | 1.0 | 0.5 |  |
| G | 55 | 1.15 | 1.78 | G | 55 | 1.2 | 0.5 | K | | 55 | 0.9 | 1.3 |  |
| G | 56 | 1.15 | 1.12 | B | 56 | 1.1 | 0.4 | G | | 56 | 0.5 | 0.3 |  |
| A | 57 | 0.35 | 0.58 | A | 57 | 2.1 | 2.1 | A | | 57 | 1.5 | 1.1 |  |
| A | 58 | 1.44 | 0.78 | A | 58 | 1.2 | 0.8 | A | | 58 | 1.3 | 1.9 |  |
| A | 59 | 0.31 | 0.84 | A | 59 | 0.8 | 0.5 | A | | 59 | 1.7 | 0.9 |  |
| A | 60 | 1.35 | 1.08 | A | 60 | 2.3 | 2.1 | A | | 60 | 1.4 | 1.2 |  |
| A | 61 | 0.09 | 0.45 | A | 61 | 0.6 | 0.6 | A | | 61 | 0.0 | 0.2 |  |
| A | 62 | 1.05 | 0.86 | A | 62 | 0.4 | 1.1 | H | | 62 | 1.6 | 1.8 |  |
| A | 63 | 0.15 | 0.14 | A | 63 | 0.0 | 0.1 | H | | 63 | 1.3 | 1.7 |  |
| A | 64 | 1.03 | 1.11 | A | 64 | 0.2 | 0.1 | A | | 64 | 0.8 | 1.4 |  |
| A | 65 | 1.21 | 1.66 | A | 65 | 0.6 | 0.4 | A | | 65 | 0.2 | 0.3 |  |
| A | 66 | 2.80 | 1.01 | H | 66 | 1.2 | 0.7 | H | | 66 | 1.1 | 0.3 |  |
| A | 67 | 0.75 | 0.38 | H | 67 | 0.7 | 1.2 | H | | 67 | 0.8 | 0.1 |  |
| A | 68 | 0.70 | 1.04 | A | 68 | 1.9 | 2.3 | H | | 68 | 1.2 | 0.7 |  |
| A | 69 | 0.79 | 0.86 | F | 69 | 1.3 | 0.3 | H | | 69 | 0.5 | 0.9 |  |
| A | 70 | 2.19 | 2.59 | F | 70 | 0.3 | 0.4 | H | | 70 | 0.7 | 1.0 |  |
| F | 71 | 0.07 | 0.33 | F | 71 | 0.4 | 0.4 | F | | 71 | 0.3 | 0.2 |  |
| F | 72 | 1.80 | 2.59 | F | 72 | 1.2 | 1.9 | F | | 72 | 1.9 | 1.5 |  |
| A | 73 | 0.82 | 1.13 | K | 73 | 1.7 | 1.2 | H | | 73 | 1.0 | 1.2 |  |
| A | 74 | 0.08 | 0.68 | H | 74 | 1.4 | 1.4 | H | | 74 | 1.0 | 1.4 |  |
| A | 75 | 0.44 | 0.35 | H | 75 | 0.5 | 0.4 | H | | 75 | 0.3 | 1.2 |  |
| A | 76 | 2.44 | 2.47 | H | 76 | 1.0 | 1.1 | H | | 76 | 1.7 | 0.7 |  |
| F | 77 | 1.26 | 1.60 | F | 77 | 0.7 | 0.4 | H | | 77 | 0.3 | 1.4 |  |
| H | 78 | 1.27 | 1.95 | K | 78 | 0.9 | 0.3 | K | | 78 | 1.1 | 1.3 |  |
| H | 79 | 0.94 | 1.41 | K | 79 | 0.7 | 1.4 | K | | 79 | 0.1 | 0.4 |  |
| H | 80 | 1.20 | 1.88 | K | 80 | 1.1 | 1.0 | H | | 80 | 0.7 | 0.5 |  |
| H | 81 | 0.10 | 0.74 | K | 81 | 1.4 | 1.8 | H | | 81 | 1.6 | 1.0 |  |
| G | 82 | 1.35 | 1.27 | G | 82 | 1.8 | 2.4 | K | | 82 | 1.7 | 1.2 |  |
| H | 83 | 0.62 | 0.68 | K | 83 | 0.6 | 0.3 | K | | 83 | 1.1 | 2.3 |  |
| H | 84 | 1.22 | 1.11 | K | 84 | 0.7 | 0.9 | K | | 84 | 1.0 | 1.0 |  |
| G | 85 | 1.28 | 1.09 | G | 85 | 1.7 | 2.0 | G | | 85 | 1.0 | 1.8 |  |
| G | 86 | 1.36 | 2.05 | G | 86 | 0.8 | 0.7 | K | | 86 | 1.2 | 0.3 |  |
| G | 87 | 0.73 | 0.83 | G | 87 | 1.5 | 1.0 | K | | 87 | 0.7 | 1.1 |  |
| G | 88 | 1.54 | 2.84 | G | 88 | 1.2 | 2.3 | K | | 88 | 0.3 | 0.1 |  |
| G | 89 | 1.82 | 0.98 | B | 89 | 0.1 | 0.4 | G | | 89 | 1.8 | 1.4 |  |
| G | 90 | 0.82 | 0.75 | B | 90 | 1.4 | 1.0 | G | | 90 | 0.1 | 0.3 |  |
| G | 91 | 0.13 | 0.25 | G | 91 | 0.3 | 0.2 | G | | 91 | 1.6 | 0.7 |  |
| B | 92 | 1.97 | 2.44 | B | 92 | 1.0 | 1.0 | B | | 92 | 1.4 | 1.7 |  |
| B | 93 | 1.12 | 0.20 | B | 93 | 1.2 | 0.4 | B | | 93 | 0.9 | 1.0 |  |
| B | 94 | 0.51 | 0.41 | B | 94 | 0.4 | 0.3 | B | | 94 | 0.4 | 0.1 |  |
| G | 95 | 1.86 | 0.95 | B | 95 | 0.4 | 0.5 | G | | 95 | 0.4 | 1.2 |  |
| B | 96 | 0.61 | 0.58 | B | 96 | 1.0 | 1.9 | B | | 96 | 0.4 | 0.6 |  |
| D | 97 | 0.63 | 0.47 | D | 97 | 0.6 | 0.9 | D | | 97 | 1.8 | 1.6 |  |
| G | 98 | 1.79 | 1.78 | B | 98 | 0.7 | 0.9 | G | | 98 | 1.1 | 0.1 |  |
| G | 99 | 0.63 | 0.47 | B | 99 | 1.2 | 0.7 | G | | 99 | 1.8 | 2.2 |  |
| G | 100 | 1.61 | 1.75 | B | 100 | 1.5 | 0.8 | C | | 100 | 0.2 | 0.5 |  |
| B | 101 | 0.79 | 0.59 | B | 101 | 2.4 | 2.4 | B | | 101 | 1.2 | 1.1 |  |
| B | 102 | 0.13 | 0.40 | B | 102 | 0.8 | 2.3 | B | | 102 | 0.8 | 0.6 |  |
| B | 103 | 1.65 | 2.36 | B | 103 | 0.6 | 0.8 | B | | 103 | 0.9 | 1.3 |  |
| B | 104 | 0.91 | 0.53 | B | 104 | 0.7 | 0.9 | C | | 104 | 0.5 | 0.1 |  |
| C | 105 | 0.47 | 1.26 | B | 105 | 2.1 | 2.5 | C | | 105 | 1.4 | 1.3 |  |
| B | 106 | 1.08 | 1.27 | B | 106 | 2.0 | 2.5 | B | | 106 | 1.9 | 1.5 |  |
| B | 107 | 0.79 | 1.45 | B | 107 | 0.7 | 1.6 | B | | 107 | 0.7 | 0.5 |  |
| B | 108 | 0.22 | 0.55 | B | 108 | 0.5 | 0.7 | B | | 108 | 2.2 | 1.3 |  |
| B | 109 | 0.07 | 0.18 | B | 109 | 0.3 | 0.2 | B | | 109 | 0.3 | 0.4 |  |
| C | 110 | 1.78 | 0.37 | B | 110 | 2.5 | 0.8 | C | | 110 | 3.0 | 1.7 |  |
| B | 111 | 2.38 | 2.57 | B | 111 | 0.2 | 0.3 | B | | 111 | 0.9 | 1.7 |  |
| B | 112 | 1.27 | 1.36 | B | 112 | 0.8 | 0.7 | B | | 112 | 1.1 | 1.5 |  |
| B | 113 | 1.21 | 0.84 | B | 113 | 0.3 | 0.1 | B | | 113 | 0.9 | 1.1 |  |
| B | 114 | 1.28 | 0.28 | C | 114 | 2.7 | 1.8 | B | | 114 | 0.2 | 0.8 |  |
| D | 115 | 0.55 | 0.57 | D | 115 | 1.4 | 1.7 | D | | 115 | 2.9 | 1.3 |  |
| D | 116 | 0.83 | 1.44 | D | 116 | 1.3 | 1.1 | D | | 116 | 0.4 | 0.6 |  |
| C | 117 | 1.75 | 0.64 | C | 117 | 0.7 | 0.3 | C | | 117 | 0.4 | 0.7 |  |
| D | 118 | 0.92 | 0.50 | C | 118 | 1.7 | 2.2 | C | | 118 | 0.3 | 0.5 |  |
| D | 119 | 0.12 | 0.13 | D | 119 | 1.9 | 1.7 | D | | 119 | 1.3 | 1.9 |  |
| D | 120 | 1.93 | 1.60 | D | 120 | 0.5 | 0.5 | D | | 120 | 0.7 | 0.6 |  |
| D | 121 | 1.79 | 2.18 | D | 121 | 0.2 | 0.5 | D | | 121 | 1.5 | 1.9 |  |

Supplementary References

1. Mallard, M. S., et al. Using a coupled lake model with WRF for dynamical downscaling, *J. Geophys. Res.: Atmos.* **119**, 7193–7208, (2014).
2. Spero, T. L., et al. The impact of incongruous lake temperatures on regional climate extremes downscaled from the CMIP5 archive using the WRF model. *J. Clim.* **29**, 839–853 (2016).
3. Herwehe, J. A., Alapaty, K., Spero, T. L., & Nolte, C. G. Increasing the credibility of regional climate simulations by introducing subgrid-scale cloud-radiation interactions. *J. Geophys. Res.: Atmos.* **119**, 5317–5330, (2014).
4. Otte, T. L., Nolte, C. G., Otte, M. J., & Bowden, J. H. Does nudging squelch the extremes in regional climate modeling? *J. Clim.* **25**, 7046–7066 (2012).
5. Hosking, J. R. M. (2019). Regional Frequency Analysis using L-Moments. R package, version 3.3. URL: https://CRAN.R-project.org/package=lmomRFA.
